# Population genetics of foxtail millet and its wild ancestor

**DOI:** 10.1186/1471-2156-11-90

**Published:** 2010-10-11

**Authors:** Chunfang Wang, Jinfeng Chen, Hui Zhi, Lu Yang, Wei Li, Yongfang Wang, Haiquan Li, Baohua Zhao, Mingsheng Chen, Xianmin Diao

**Affiliations:** 1 The National Key Facility for Crop Gene Resources and Genetic Improvement (NFCRI), Institute of Crop Science, the Chinese Academy of Agricultural Sciences (CAAS), Beijing 100081, China; 2 Institute of Millet Crops, Hebei Academy of Agricultural and Forestry Science, Shijiazhuang 050031, China; 3 State Key Laboratory of Plant Genomics, Institute of Genetics and Developmental Biology, Chinese Academy of Sciences, Beijing 100101, China; 4 College of Life Sciences, Hebei Normal University, Shijiazhuang 050012, China

## Abstract

**Background:**

Foxtail millet (*Setaria italica *(L.) P. Beauv.), one of the most ancient domesticated crops, is becoming a model system for studying biofuel crops and comparative genomics in the grasses. However, knowledge on the level of genetic diversity and linkage disequilibrium (LD) is very limited in this crop and its wild ancestor, green foxtail (*Setaria viridis *(L.) P. Beauv.). Such information would help us to understand the domestication process of cultivated species and will allow further research in these species, including association mapping and identification of agricultural significant genes involved in domestication.

**Results:**

In this study, we surveyed DNA sequence for nine loci across 50 accessions of cultivated foxtail millet and 34 of its wild progenitor. We found a low level of genetic diversity in wild green foxtail (θ = 0.0059), θ means Watterson's estimator of θ. Despite of a 55% loss of its wild diversity, foxtail millet still harbored a considerable level of diversity (θ = 0.0027) when compared to rice and sorghum (θ = 0.0024 and 0.0034, respectively). The level of LD in the domesticated foxtail millet extends to 1 kb, while it decayed rapidly to a negligible level within 150 bp in wild green foxtail. Using coalescent simulation, we estimated the bottleneck severity at k = 0.6095 when ρ/θ = 1. These results indicated that the domestication bottleneck of foxtail millet was more severe than that of maize but slightly less pronounced than that of rice.

**Conclusions:**

The results in this study establish a general framework for the domestication history of foxtail millet. The low level of genetic diversity and the increased level of LD in foxtail millet are mainly caused by a population bottleneck, although gene flow from foxtail millet to green foxtail is another factor that may have shaped the pattern of genetic diversity of these two related gene pools. The knowledge provided in this study will benefit future population based studies in foxtail millet.

## Background

Plant domestication, which began approximately 10,000 years ago, is the most crucial development in human history [1]. Domesticated crops provide most of our food today, and provide the foundation for human civilization. Yet, only a small fraction of flowering plants were actually domesticated. It will be of great benefit for future crop breeding and improvement if we have a better understanding of the domestication process.

The evolutionary footprints left by domestication at the population level are dispersed throughout the genome. Due to population bottlenecks, a large proportion of genetic diversity is typically lost during the domestication process, as seen by a 25% reduction of diversity in maize and an 80% reduction in rice [2,3]. In some species like rice and sunflower, the transition of mating system from outcrossing to self-fertilization can further reduce the levels of genetic diversity [4,5]. Furthermore, some selected loci exhibit a more dramatic loss in genetic diversity [6]. In maize, the reduction in genetic diversity is less than 20% for presumably neutral loci like *hm1*, *hm2*, *glb1*, and *sh1*, but up to 80% for selected loci like *c1*, *ae1 *and *tb1 *[7-11]. Elevated levels of linkage disequilibrium (LD) are also observed in the genome of domesticated plants [3,6,12]. This is caused by either the domestication bottleneck and/or reduction of effective recombination rate due to self-fertilization [5]. Additionally, selection can have a similar effect on LD in the target region [13]. Studies that focus on the level and organization of genetic variation in these major crops are essential for our understanding of the process of domestication and are instructive for crop improvement or other research that is based on these population parameters, such as association mapping [2,12,14].

To study the patterns of genetic diversity within and between populations, as well as to trace the demographic history of crops and their wild relatives, multilocus surveys of population sequence data have been widely used in recent years [3,6,12,15]. Since selection acts on some but not all genes in genome, a multilocus sample increases the probability that both loci that are under selection and those that are neutrally-evolving will be sampled. Multi-locus sampling is also necessary to understand the demographic history of populations, and enables targets of natural or artificial selection to be more reliably identified. Many studies have been carried out to investigate nucleotide diversity in plants, yet only a few have been focused on their demographic history. Understanding the demographic history of populations will also help in interpretation of population genetic neutrality tests, which are based on the comparison of observed and expected polymorphism patterns under the neutral equilibrium model (NE) [16,17]. This model assumes random outcrossing and a large stable population size, assumptions which may not be valid in many domesticated crops [16]. Without a reliable knowledge of the demographic history, it is hard to interpret the statistical results of neutrality tests.

Foxtail millet (*Setaria italica *(L.) P. Beauv) has been a very important cereal since ancient times in Eurasia and has contributed greatly to human civilization both in Asia and Europe [18]. The most recent archaeological evidence demonstrates that foxtail millet is one of the most ancient crops as its domestication in China dates back to 8,700 years ago [19]. With the rapid development of maize and other modern crops, foxtail millet has gradually become a minor crop in the last 80 years, but is nonetheless still widely cultivated in Asia, Europe, North America, Australia and North Africa as grain food or forage [20]. Green foxtail (*Setaria viridis *(L.) Beauv.), a weed distributed worldwide, is the presumed wild progenitor of domesticated foxtail millet, based on cytological evidence and RAPD, AFLP, and other markers [21,22]. But the number of domestication centers and the age of domestication of foxtail millet remains controversial [23]. As revealed by earlier studies, genetic diversity is low in foxtail millet [24], yet these analyses were based on genetic markers that only represent a subset of the possible information that can be gained regarding genomic polymorphism [16]. However, research at the sequence level is very limited in foxtail millet and its close relatives. although sequencing of the foxtail millet genome is now nearly completed [25]. This will provide another cereal model system for comparative and functional genomics and model for studying other biofuel crops such as switchgrass (*Panicum virgatum*), and napiergrass (*Pennisetum purpureum*). Studies on domestication or other agricultural related characteristics are ongoing[26-28]. In this study, we conduct a multilocus analysis of nucleotide variation in foxtail millet and green foxtail to reveal the pattern of genetic diversity within and between these two species and to establish a population genetic framework for further analysis of the effects of domestication in foxtail millet.

## Results

### Nucleotide diversity and neutrality test

We collected 50 cultivated foxtail millets and 34 wild green foxtails to represent the broad diversity of these two species (Table 1). Nine loci randomly selected from the genome were used to survey genetic diversity from both sspecies (Table 2& Additional file 1, Fig s1). The alignment length of each locus after excluding gaps and missing data varied from 431 bp to 996 bp. All nine loci contained both coding and noncoding sequences. However, ninety percent of the final alignments were noncoding sequence because the primers were designed to amplify a large proportion of intron fragments. A total of 160 SNPs were found in the nine loci across the 84 accessions, with an average density of 52 bp/SNP. The wild progenitor had more SNPs (147 SNPs with a density of 43 bp/SNP) compared to the cultivars (75 SNPs with a density of 87 bp/SNP). Indel polymorphisms were infrequent across loci and most had a small size of 1 to 3 basepairs. Three large indels were present in *ADTY *(143 bp), *UPL *(112 bp) and *TIFIIF *(43) bp. These indel polymorphisms were excluded from the subsequent analysis.

**Table 1:** Plant materials used in this study.

Taxon	Accessions^a^	Origin	Code	Variety Name
*Setaria italica *ssp. *italica*				
	00014654	Gongyang, Yunnan, China	g137Yunnan	NoMiXiaoChuiNiu
	00014528	Anyang, Henan, China	g141Henan	XiaoHongGu
	00012913	Qihe, Shandong, China	g133Shandong	HongMaoGu
	00014617	Ninghai, Zhejiang, China	g136Zhejiang	NingHaiGuZi
	00014570	Shaanxi, China	g135Shaanxi	LaoLaiBian
	00014625	Yuanling, Hunan, China	n793Hunan	HuangBangTou
	00022285	Leshan, Sichuan, China	n1549Sichuan	LeShanBaiNuo
	00005252	Fanshi, Shanxi, China	n286Shanxi	LiuLengGu
	00001532	Kangping, Liaoning, China	n107Liaoning	HeiNianGu
	00000077	Nenjiang, Heilongjiang, China	n6Heilongjiang	CaoPiYiDaoBaQi
	00000158	Baiquan, Heilongjiang, China	n9Heilongjiang	FuoDingZhu
	00021705	Gansu, China	n1473Gansu	DaLiangZhouGu
	00018757	Hualong, Qinghai, China	n1187Qinghai	XiaoHongGu
	00018782	Minhe, Qinghai, China	n1199Qinghai	BaoMaoGu
	00018783	Minhe, Qinghai, China	n1200Qinghai	DaliangGu
	00014609	Xinjiang, China	n779Xinjiang	EminGuZi
	00014612	Xinjiang, China	n782Xinjiang	ShaWanGuzi
	00018751	Guyuan, Ningxia, China	n1185Ningxia	XiaoMiaoGu
	PI 433458	Taiwan, China	g45Taiwan	Megalaoud
	PI 433396	Taiwan, China	g50Taiwan	Pagarugareano
	PI 433465	Taiwan, China	g54Taiwan	Balahigh
	PI 433481	Taiwan, China	g121Taiwan	Ishsumsum
	00014695	Japan	n822Japan	ZhaoHeNuo
	00014706	Japan	n824Japan	LiuShiRi
	PI 464241	Bihar, India	g27India	I.Se 83
	PI 454359	Maharashtra, India	g32India	I.Se 210-B
	PI 464157	Andhra Pradesh, India	g35India	I.Se 1
	PI 464287	Kerala, India	g36India	I.Se 141
	PI 464457	Punjab, India	g39India	I.Se 304
	PI 427256	Nepal	g16Nepal	
	PI 269972	Pakistan	g21Pakistan	
	PI 251395	Iran	g22Iran	BAJRA
	PI 220634	Afghanistan	g18Afghanistan	
	PI 473601	Lebanon	g64Lebanon	
	Ames 21521	Kazakhstan	g14Kazakhstan	VYSOKOROSKY
	PI 177543	Turkey	g5Turkey	KUMDARI
	00014986	Nertherland	n836Nertherland	Kraftborn
	00015029	German	n840German	Set64/82
	PI 442551	Belgium	g51Belgium	
	PI 283988	Spain	g1Spain	
	PI 464567	Switzerland	g68Switzerland	I.Se 663
	PI 290459	Hungary	g3Hungery	
	00014980	Romania	g139Romania	Romania 1
	00014968	Russian	g132Russian	
	PI 464544	Ethiopia	g28Ethiopia	I.Se 410-B
	PI 209909	South Africa	g9SouthAfrican	
	PI 517051	Morocco	g72Morocco	GR848
	00015040	USA	n842USA	
	00015042	USA	n843USA	
	00015044	USA	n844USA	
*Setaria italica *ssp. *viridis*				
	8199W64	England	q140England	
	8200W65	France	q131France	
	8201W66	German	q94German	
	8012Q10	Bashang, Hebei, China	q90Hebei	
	8019Q17	Baoding, Hebei, China	q91Hebei	
	8003Q03	Qinghai, China	q92Qinghai	
	8044Q35	Chifeng, Neimeng, China	q88Neimeng	
	8045Q36	Heilongjiang, China	q95Heilongjiang	
	8049Q39	Liaoning, China	q96Liaoning	
	8004Q04	Lanzhou, Gansu, China	q93Gansu	
	8058Q46	Changzhi, Shanxi, China	q143Shanxi	
	8005Q05	Xinjiang, China	q89Xinjiang	
	8066Q54	Shandong, China	q87Shandong	
	8009Q7-1	Kunming, Yunnan, China	q142Yunnan	
	GZ001	Guangzhou, China	qGuangzhou	
	UC9001	Uzbekistan	qUzbekistan	
	UC9012	Japan	qJapan	
	ZH001	Zhejiang, China	qZhejiang1	
	Ames 21519	Russian	q77Russian	
	Ames 21520	Russian	q134Russian	
	PI 202407	Chile	q80Chile	
	PI 204624	Turkey	q75Turkey	
	PI 204625	Turkey	q76Turkey	
	PI 204628	Turkey	q81Turkey	
	PI 204727	Turkey	q83Turkey	
	PI 204730	Turkey	q86Turkey	
	PI 212625	Afgnanistan	q73Afghanistan	
	PI 221960	Afgnanistan	q74Afghanistan	
	PI 223677	Iran	q78Iran	
	PI 230134	Iran	q79Iran	
	PI 230135	Iran	q85Iran	
	PI 408810	Changchun, Jilin, China	q138Jilin	
	PI 408811	Shaanxi, China	q84Shannxi	
	Pi 442553	Belgium	q144Belgium	

^a ^Accession numbers started with PI or Ames were from the National Germplasm Resources Laboratory, Beltsville, Maryland, USA;

Others samples were from Chinese National Germplasm Bank in Institute of Crop Sciences, Chinese Academy of Agricultural Sciences.

**Table 2:** Summary of the genes surveyed and the primer sequences used in the study.

Gene Name	Rice/Millet	Putative function	Primers
*DACP*	LOC_Os01g21160.2/EC612491	Dhydrolipoyllysine-residue acetyltransferase	F:5-3'ACACTTTCCTTCCGTTCCTCAT
			R:5-3'TGGTGCCATCTTCAATATCTGC
*SIGT*	LOC_Os01g09120.2/EC613421	Signal transducer/two-component sensor molecule	F:5-3' ATCCCAGCACTCAGTTCTTCAT
			R:5-3' AGACTCTGCAGTTACAGCCCA
*ADTY*	LOC_Os02g56550.1/EC612081	ATP-dependent transporter YFL028C	F:5'-3' TCCACTACAAGGCGATTTCT
			R:5'-3' ATCCATTCCGGTCACAACAT
*PP2C*	LOC_Os03g60650.1/EC612551	Catalytic/protein phosphatase type 2C	F:5'-3' TGTGAAGGGCTCGCTTAAG
			R:5'-3' GACGACCCAACGTAATCTATTC
*SPS1*	LOC_Os08g20660.2/EC612114	Sucrose-phosphate synthase 1	F:5-3' TTGGCTTCTCGCTCACAGG
			R:5-3' CACCTCCAAGCAAACCTTCA
*UPL*	LOC_Os10g41360.1/EC611973	Ubiquitin-protein ligase	F:5-3' AGTGGTGCTGAGATTGGTAGA
			R:5-3' GATGGTGCTCCAAGTTCCTG
*TIFIIF*	LOC_Os10g10990.3/EC613446	Transcription initiation factor IIF, alpha subunit	F:5-3' TCTTCTTGCTGTGGCTCCAG
			R:5-3' AAGGACGACGTAGTTGTGGC
*TRAN*	LOC_Os11g37980.1/EC612732	Transferase, transferring glycosyl groups	F:5-3' TATGAAGGGTAAAGTAATTGCTGC
			R:5-3' GGGTTTGAGTTTCCCGCTGT
*MDEH*	LOC_Os12g43630.1/EC613245	Malate dehydrogenase, glyoxysomal precursor	F:5-3' CTTGGGCCATTGAATGAGTT
			R:5-3' GACGCCCTTCTGGATACTCT

The nucleotide diversity of the nine loci for each species is summarized in Table 3. For both θ and π, the values for each locus were slightly lower than values for silent sites, presumably due to strong functional constraint in coding regions. Considering individual loci, the most variable gene was *MDEH *with a mean of 0.01 for θ_sil _across all accessions. Compared to *MDEH*, *PP2C *was the least variable site with a value of only 0.0016 for θ_sil_. At the taxon level, the diversity of wild green foxtail (mean θ_sil _0.0059) was much higher than the domesticated foxtail millet (mean θ_sil _0.0027). On average, the cultivars lost 55% of the diversity harbored by the wild progenitor during the domestication process. Some loci in cultivars, such as *ADTY *and *PP2C*, lost more than 86% and 75% of their diversity in the wild progenitor. Under the assumption of a neutral equilibrium model, the population silent diversity (θ_sil_) and population divergence (Ks) should be correlated with each other across the loci. We calculated the Pearson correlation between them to test if both species were under neutral evolution. The correlation for wild species is high and significant (pearson cor = 0.93, p value = 0.0001), whereas it is not significant for the cultivar (pearson cor = 0.32, p = 0.19). When excluding the most diverged locus *ADTY*, significant positive correlations were found in both wild (pearson cor = 0.89, p = 0.002) and cultivars species (pearson cor = 0.69, p = 0.03). The significant correlations suggested that most of loci were under neutral evolution in both species, except for *ADTY *in cultivars. This locus may be under directional selection or influenced by a selective sweep on neighboring loci.

**Table 3:** Summary of nucleotide diversity and neutrality tests

Loci	Length^a^	Silent	Accessions^b^	S	π	πsilent	Θ	θslient	D	D*	F*	ρ	Rm	Hap	Hdiv
*Setaria italica *ssp. *viridis*															
*DACP*	1014	944.833	29	27	0.0062	0.0067	0.0068	0.0073	-0.3118	-0.4487	-0.4763	0.0002	0	13	0.877
*SIGT*	732	681.833	29	10	0.0014	0.0015	0.0035	0.0037	-1.9503*	-2.8589*	-3.0169*	0.0052	0	8	0.643
*ADTY*	997	892.011	31	41	0.01	0.0111	0.0105	0.0112	-0.1808	-0.5215	-0.5215	0.0076	0	17	0.931
*PP2C*	815	815	33	8	0.0021	0.0021	0.0024	0.0024	-0.3792	-0.6279	-0.6449	0.1034	1	8	0.799
*SPS1*	716	716	31	10	0.0018	0.0018	0.0035	0.0035	-1.5505	-1.3113	-1.6212	0	0	7	0.452
*UPL*	656	636.667	32	9	0.0024	0.0025	0.0034	0.0035	-0.8911	-1.0025	-1.1326	0.0027	0	6	0.609
*TIFIIF*	569	521	29	9	0.0025	0.0027	0.004	0.0044	-1.1863	-0.9376	-1.1834	0.0253	0	9	0.717
*TRAN*	440	195.187	32	14	0.0062	0.0063	0.0085	0.0085	-0.8844	-0.4649	-0.7027	1.1713	0	12	0.891
*MDEH*	525	525	31	19	0.0042	0.0042	0.0091	0.0091	-1.8459*	-2.6617*	-2.8235*	0.0315	0	14	0.819
Average	718	658.614	30.8	16.3	0.004	0.0043	0.0057	0.0059	-1.02	-1.2039	-1.347	0.1497	0.1	10.4	0.748
*Setaria italica *ssp. *italica*															
*DACP*	964	896	46	19	0.0035	0.0038	0.0045	0.0048	-0.6808	0.9081	0.4346	0	0	7	0.633
*SIGT*	767	715.667	48	7	0.0012	0.0013	0.0021	0.0022	-1.0991	-0.3006	-0.6509	0	0	4	0.264
*ADTY*	1005	900	50	6	0.0002	0.0003	0.0013	0.0015	-2.0897*	-3.9817*	-3.9678*	0	0	3	0.079
*PP2C*	760	760	47	2	0.0007	0.0007	0.0006	0.0006	0.3793	-0.8753	-0.5879	Na	0	3	0.528
*SPS1*	695	695	46	4	0.0016	0.0016	0.0013	0.0013	0.476	1.0127	0.9907	0.0041	0	3	0.518
*UPL*	761	734.333	48	6	0.0012	0.0012	0.0018	0.0018	-0.8501	-0.5283	-0.7377	0.0077	0	7	0.54
*TIFIIF*	624	556.833	44	6	0.0019	0.002	0.0022	0.0017	-0.3342	-2.1726	-1.8723	0.0093	0	5	0.577
*TRAN*	435	188.543	49	10	0.0042	0.0056	0.0052	0.0036	-0.5283	-0.4341	-0.5478	0.0219	1	9	0.743
*MDEH*	549	549	43	15	0.0039	0.0039	0.0067	0.0067	-1.3336	0.7496	0.0648	0.2204	1	14	0.886
Average	729	666.153	46.8	8.3	0.0021	0.0023	0.0029	0.0027	-0.6734	-0.6247	-0.7638	0.0329	0.2	6.1	0.529

Note: Length, alignment length; Silent, alignment length on silent site; Aceessions, number of accessions sequenced for each locus; S, the number of segregating sites; **π**, average number of nucleatide difference per site between 2 sequence; **π silent**, **π **on silent sites; **θ**, the watterson estimator of population mutation rate **θ**; **θ silent**, **θ **on silent sites; ***D***, Tajima's *D*; D* and F*, D* and F* of Fu and li; **ρ**, the population recombination rate; ***R*_M_**, the minimum number of recombination events; **Hap**, number of haplotype; **Hdiv**, haplotype diversity.

**Figure 1:**
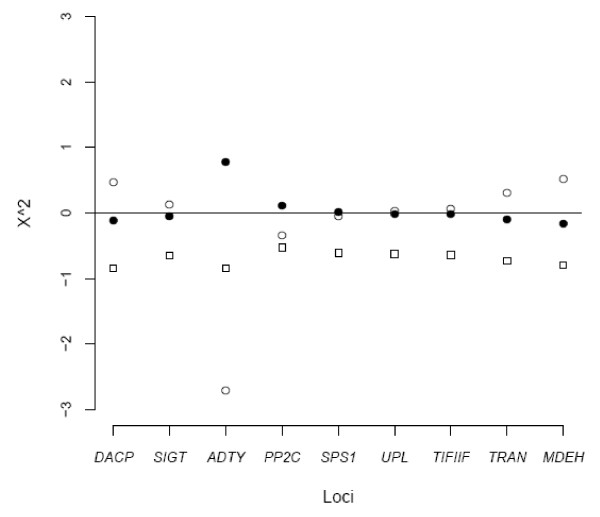
**Summary of multilocus HKA test**. Blank circles stand for deviations of foxtail millet. Solid circles stand for deviations of green foxtail. Squares stand for deviations of divergence between foxtail millet and green foxtail.

We used a series of neutrality tests to determine the fit of our data to a neutral equilibrium model. Tajima's D and Fu and Li's D* and F* were used to examine the allele frequency spectrum in polymorphism data for each locus. In the wild population, all the loci showed a negative value for D, D* and F*. Two loci, *SIGT *and *MDEH*, had a significant negative value for both tests. However, loci in the cultivars exhibited positive values or less negative values than the wild species for both tests except for *DACP *and *ADTY*. The results were unsurprising since we expected D to be higher when the species had experienced a recent population bottleneck[29]. The loss of low frequency alleles during the bottleneck process will increase the D for cultivars. On the other hand, a significant negative D value means the locus has an excess of low frequency variants due to population size expansion and/or purifying or directional selection. We also used a multilocus HKA test to determine whether the level of polymorphism and divergence were correlated across the loci. A significant result was found when we used all nine loci (X^2 = 12.26, p < 0.007). As shown in Figure 1, locus *ADTY *contributed a large proportion to the overall deviation. Further analysis by removing *ADTY *showed that no significant result was found across the loci (X^2 = 6.92, p < 0.21).

### Population divergence

The level of population differentiation of the two related species was examined by the values of Fst and shared, fixed and unique polymorphisms in the two species, as well as the phylogenetic relationship of these accessions. Fst varied from locus to locus with a mean of 0.1536 (Table 4). Although some loci, such as *ADTY *and *UPL*, had Fst values over 0.40, the remaining loci were differentiated at a very low level with Fst values between 0.0254-0.1546. No fixed differences were detected between the cultivars and their wild relatives. This was consistent with a low divergence level due to the short history of domestication. However, a high proportion of shared polymorphisms were observed, particularly for the cultivars. The domesticated foxtail millet shared almost 75% of its polymorphism with green foxtail on average, whereas the proportion for green foxtail was 36%. Unique polymorphisms were present in both species, but the number was much less in cultivars compared to the wild species. Due to the nature of the domestication process, we expected that the cultivars had lost much of their diversity compared to its wild progenitor (55%). The unique polymorphisms in the cultivars suggested that new mutations occurred after domestication or that the wild gene pool was insufficiently sampled.

**Table 4:** Summary statistics of population differentiation

Loci	S_Shared_	S_Fixed_	S_wild specific_	S_cultivar specific_	Fst
*DACP*	18	0	9	1	0.0254
*SIGT*	5	0	4	1	0.0289
*ADTY*	4	0	37	1	0.4085
*PP2C*	2	0	4	0	0.0933
*SPS1*	2	0	8	1	0.1546
*UPL*	1	0	8	4	0.4082
*TIFIIF*	3	0	6	1	0.1002
*TRAN*	7	0	8	3	0.1075
*MDEH*	10	0	9	6	0.0556
Average	5.8	0	10.3	2	0.1536

### Linkage disequilibrium

**Figure 2:**
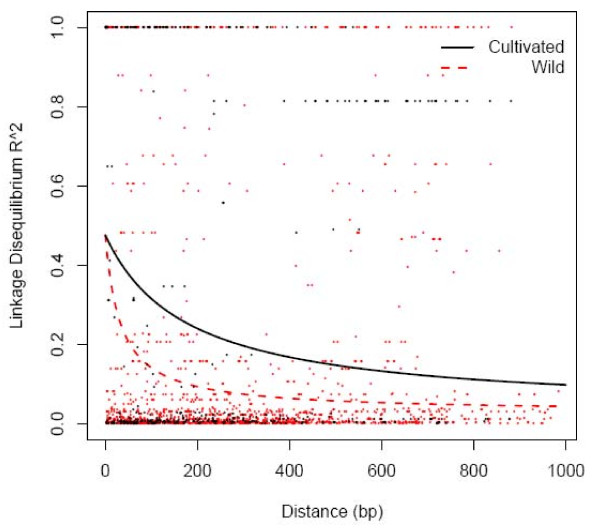
**Plots of squared allele-frequency correlations along with the distance between pairs of SNP across nine loci**.

The level of linkage disequilibrium measured as squared allele-frequency correlations were plotted against the distance between pairwise SNPs (Figure 2). The regression curves show that the decay of linkage disequilibrium along the distance for the cultivars was much slower than for the wild. The expected value of for wild green foxtail dropped rapidly to 0.1 within 150 bp, whereas a much higher level (> 0.1) extended to 1000 bp in foxtail millet. Comparison of the population recombination rate, ρ, in the two species implied that recombination was more frequent in the wild species (mean = 0.1497) than that in the domesticated species (mean = 0.0329). Together with the data of haplotype number and haplotype diversity (table 3), the cultivar foxtail millet showed increased linkage disequilibrium level compared to the wild progenitor.

### Bayesian estimate of population mutation rate θ and recombination rate/mutation rate ρ/θ

**Figure 3:**
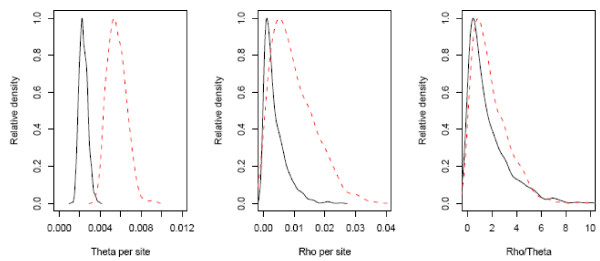
**Posterior distributions of θ, ρ and ρ/θ as estimated using approximate Bayesian approach in foxtail millet (solid line) and green foxtail(dashed line)**.

Under the standard neutral model, we drew the prior distribution of θ and ρ from uniform distribution within intervals 0-0.03 and 0-0.15. Using rejection algorithm, we obtained 1000 samples from simulations. The posterior distribution of θ, ρ and ρ/θ are shown in Figure 3. Compared with the mean values calculated from the sequence data, values estimated by simulations were quite similar for θ in both domesticated (0.0022) and wild species (0.0053). However, the maximum posterior estimates of ρ were not as large as observed data (ρ = 0.001, ρ/θ = 0.43 for the domesticated and ρ = 0.005 and ρ/θ = 0.836 for the wild). Since the sequence used in this study were short, recombination that could be detected in such a short distance would be rare. The method used for calculating recombination rates here may not give correct values. We also found variations among loci for the values of ρ (0-1.1713 for the cultivated and 0-0.2204 for the wild). This method may over-estimate ρ for some loci. We used a grid of values 0, 1, 2, 5, 10 for ρ/θ in the later analysis, since previous studies showed that the level of recombination had influence on posterior estimation of parameters [30].

### Bayesian inference of bottleneck process

**Figure 4:**
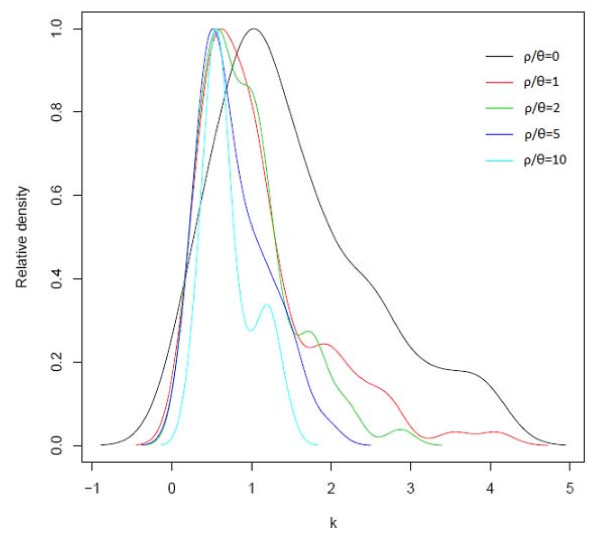
**Posterior distributions of population bottleneck severity k estimated using approximate Bayesian approach for a grid of ρ/θ = 0, 1, 2, 5, 10**.

**Figure 5:**
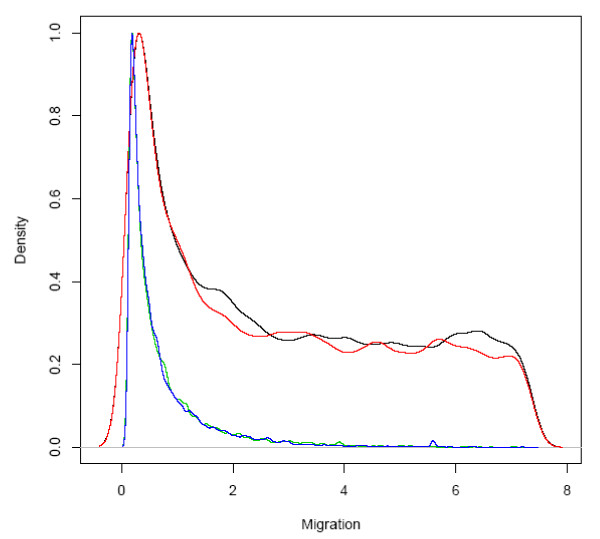
**Posterior distributions of migrations between foxtail millet and green foxtail using a Markov Chain Monte Carlo (MCMC) method**. Two independent runs were run to assess convergence of estimation, where red and black lines stand for the migrations from foxtail millet to green foxtail each generation and blue and green lines stand for the reverse process.

Domestication process can be modeled by coalescent simulation using a simple bottleneck model. The model has been described in Maize, Rice, Wheat and other crops, although for species such as Asian rice the domestication scenario may be more complex[2,3,6,15]. In the bottleneck model, it was assumed that the domesticated species experienced a reduction in population size in the initial of domestication state and then the population size increased after the domesticated species were wildly distributed. We can use this model to infer the domestication process based on population structure and other known information. Upon the assumption of the domestication model, a series of coalescent simulations were done to infer the parameters of the bottleneck model. The parameters that need to be estimated were drawn from a uniform distribution with a specific interval (t2 = 5000-15000, d = 100-3000, k = 0.1-10, m12 = 0-100, m21 = 0-100) (table 3). We did simulations for ρ/θ = 0, 1, 2, 5, 10, respectively. As the acceptance rates for simulations of ρ/θ > = 2 were very low, we used a larger interval (60%) for the rejection algorithm in these simulations. For each simulation, we collected 10,000 samples using the rejection algorithm with summary statistics of the wild species. Then the samples were used to fit with summary statistics of the cultivated species. The number of fitted samples used for posterior prediction varied from 702 for ρ/θ = 1 to 2571 for ρ/θ = 2. The posterior distribution of t2, d, M12 and M21 showed no large peak. However, the posterior distribution of the bottleneck intensity k did show a clear peak and depended on the ratio of ρ/θ (Figure 4). The domestication bottleneck was more severe when ρ/θ changed to large values. Since the ratio of ρ/θ estimated by Bayesian estimation in this study was 0.836, the severity of the bottleneck for foxtail millet should be similar to that of ρ/θ = 1 (0.6095). If the ratio of ρ/θ was underestimated, the domestication process would be much more intense. To further estimate the rate of migration between the two species, we employed a Markov Chain Monte Carlo method as implemented in MIMAR [31]. We ran 1.1e7 steps with 1e6 burnin steps and considered convergence was reached when the posterior distribution of two independent chains were similar (Figure 5). The maximum estimated migration from cultivar to wild is 0.3174, whereas the reverse process was 0.1712.

## Discussion

### Sequence diversity

**Figure 6:**
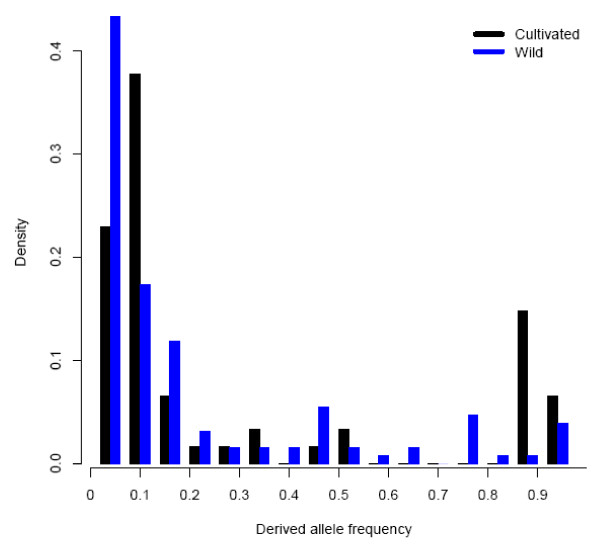
**Derived allele frequency spectra for cultivated foxtail millet and wild green foxtail**.

Previous studies based on isozymes and DNA markers showed a high level of genetic diversity in foxtail millet [32,33]. However, diversity at the DNA sequence level of foxtail millet was not well documented and comparisons with other crops, especially cereals, was absent. In this study, nine loci were surveyed to investigate the genetic diversity in foxtail millet and its wild progenitor green foxtail at the DNA sequence level. We found a low level of nucleotide variation in both foxtail millet and green foxtail as compared with other domesticated crops and their wild relatives, such as sunflower, barley, maize and rice [3,6,12,34]. The silent nucleotide variation for green foxtail estimated here was 0.0059, which was lower than most of these wild crop relatives but higher than wild soybean. Compared to other species, nucleotide variation indicated by θ_sil _was higher in wild maize *Zea mays *ssp. *parviglumis *(0.0247), wild sunflower *Helianthus annuus *(0.0234), etc [3,6,12,34]. An exception was found in wild soybean *Glycine soja*, which had a θ_sil _as low as 0.00235 [35]. Further investigation of these wild relatives showed that most wild species with a higher nucleotide variation have an outcrossing mating system, while nucleotide variation was lower for inbreeding species, such as wild barley and wild soybean [34,36,37]. Green foxtail, as well as foxtail millet, are self-pollinated plant that have a 0.3% to 4% outcrossing rate in natural conditions [38,39]. Lower levels of nucleotide variation in these wild species is expected by their mating system, although the samples collected in this study may also influence the estimation. This is because some wild accessions may not represent a local original wild species but a weedy form that derived from the gene flow between the cultivars and their wild relatives. However, the underestimation influenced by sampling may have been very limited because several accessions were collected in the areas where a weedy form was recognized. The low level of genetic diversity of wild green foxtail might be caused mainly by its high inbreeding rate and/or other demographic factors, particularly changes in population size. Consistent with its wild relatives, foxtail millet also showed a lower level of silent nucleotide variation (0.0027). It was much lower than that of maize (0.0149) or sunflower (0.0072), but similar to that of rice (0.0024) and sorghum (0.0034) [3,6,12,40]. The domestication of maize, barley, soybean, and foxtail millet did not involve changes in mating systems. However, the mating system was changed from outcrossing to inbreeding in rice and sunflower [3,12]. Based on this information, we can conclude that the level of genetic diversity of foxtail millet, which maintained 45% of its wild diversity, was mainly a result of its change in population size during domestication process, followed by mutation accumulated after divergence. To further explore the impact of domestication on genetic diversity, we used Tajima's D test to detect the change of SNP frequency in the species after domestication. As indicated by Tajima' test, D values of most loci in domesticated species were higher than those of the wild species, but only two of them had a positive D value. The increase of D values in the domesticated species is likely because low frequency alleles were preferentially lost during the domestication bottleneck. Detailed site frequency spectra are shown in Figure 6. We detected an excess in both low and high frequency alleles in the domesticated species compared to the wild one. The excess of high frequency variants in the domesticated species was also found in domesticated Asian rice [41]. Taking into account the higher level of LD in the domesticated species, this may mean that artificial selection active during the domestication process may have extended over a long distance. This phenomenon has been observed for *tb*1 in maize and *waxy *in rice, that the influence of directional selection on certain loci had a large effect on regions around them [8,42].

### Linkage disequilibrium

Several studies that focus on the domestication of crops indicated that there was an increased level of linkage disequilibrium in domesticated species as compared to their wild relatives [3,12,43]. With the bottleneck effect of domestication, the genome-wide level of nonrandom association tends to increase. In some loci that were targeted by artificial selection, LD could extend to a long distance up to 100 kb, such as *tb*1 in maize and *waxy *in rice [8,42]. In addition to the influence of population size and direction selection, mating system was also shown to affect LD level [16]. In selfing *Arabidopsis thaliana*, LD can persist for 250 kb. However, in outcrossing maize, LD declined to a very low level of less than 1 kb [43,44]. Using the same set of sequences, it was shown that the LD level in *O. nivara *was slightly higher than that in *O. rufipogon*, which displayed a higher rate of outcrossing compared to *O. nivara *[3]. In this study, the higher level of LD observed in foxtail millet compared with green foxtail was similar to what has been observed in rice and sunflower [3,12]. In wild species, which were shown to have a higher outcrossing rate, the LD level declined rapidly within 100-200 bp. In contrast, LD in domesticated foxtail millet extended to 1000 bp. Based on the fact that the mating system did not change after domestication of foxtail millet, and that the influences of directional selection was likely focused on a small proportion of local genomic regions, we suggest that the increased level of LD in the cultivated foxtail millet was mainly due to the change of population size during the domestication process.

### Gene flow between the domesticated and the wild

As indicated previously, foxtail millet maintained 45% of its wild diversity. The proportion was similar with that of sorghum (60-70%) and sunflower (40-59%) [12,40]. However diversity retention was higher in maize (80%) and very low in rice (10-20%) [3,16]. The retention of wild diversity is a reflection of the bottleneck intensity (such as in rice) or the mating system (such as in maize). We used coalescent simulation to infer the bottleneck severity during the domestication process. The Bayesian estimate of bottleneck severity k was 0.6095 if we set ρ/θ = 1, which is near the maximum estimate of ρ/θ. Compared with the bottleneck severity that modeled in maize (2.45) and in rice (0.2 for *japonica *and 0.5 for *indica*) [3,45], the severity estimated here was compatible with the loss of diversity from its wild relatives. We also incorporated gene flow into a simulation model. The results suggested that there were low levels of gene flow from the cultivated species to wild species, but the reverse process was even smaller. As foxtail millet and green foxtail can have low levels of cross-pollination and the hybridization between them are compatible [46], we expected that gene flow between the two species would be frequent. By intensive human selection, alleles from wild relatives to cultivated foxtail millet were mostly eliminated. But genes introgressed into the wild species might be retained at a higher level, as evidenced by many weedy types morphologically similar to foxtail millet in and around millet fields. This has became a serious problem for field management. Taken together, the population bottleneck and gene flow both contributed to the present pattern of nucleotide diversity of these two species.

### Loci under selection in the domestication

Another factor in the domestication process is human selection [16]. We intended to select nearly neutral loci in order to make inferences about the domestication process in this study. However, *ADTY *had a significant negative D value in the domesticated species. This observation, taken together with an 86% loss of diversity compared to the wild type and a large deviation in the HKA test, suggested that this locus was likely under directional selection during domestication. We used the estimated parameter of bottleneck severity to infer whether this locus was under selection. Two loci were detected to be under selection in this model, *ADTY *and *PP2C *(p-value = 0.014 and 0.008). Except for a loss of 75% of its wild diversity, other statistical tests did not show any signal of selection for *PP2C*, indicating that the simulation may have given a false positive result for this locus due to low diversity in both cultivated and wild species. Furthermore, the model used to detect selection may not be robust enough to give a result with only a few false positive genes. The locus *ADTY *may be a target gene of human selection or located in a selected region. However, excluding this locus did not affect the calculated parameters and the results of the simulation. The whole genome sequence of foxtail millet will be released very soon; and further work that focuses on whole genome analysis of genes involved in domestication will likely indicate to what extent human selection has acted in the domestication process.

## Conclusions

We found a 55% loss of diversity in foxtail millet and an increased level of LD that can be extended to 1 kb. This phenomenon is likely to be caused by the small effective population size due to a population bottleneck during domestication. Low levels of gene flow from foxtail millet to green foxtail may have been another factor that influenced the genetic diversity of these two species.

## Methods

### Plant materials and DNA sequencing

We collected 84 accessions to survey DNA sequence variation in this study, including 50 cultivated foxtail millets and 34 wild green foxtails (Table 1). Accession numbers started with PI or Ames were obtained from the National Germplasm Resources Laboratory, Beltsville, Maryland, USA; others samples were from the Chinese National Germplasm Bank in the Institute of Crop Sciences, Chinese Academy of Agricultural Sciences. The cultivated materials were sampled to represent a broad diversity of foxtail millet, in which all Chinese accessions are landraces. The wild samples were collected throughout the Eurasian continent to cover the distribution range of green foxtail where foxtail millet was claimed to have been domesticated [23,24]. All the sample seeds were planted in autoclaved soil and fresh leaves were collected to extract genomic DNA using a modified CTAB protocol.

Genomic fragments between 500 and 1500 bp were amplified and sequenced from nine unlinked loci (Table 2 and Additional file 1: figure S1). Based on the high colinearity between the genetic map of foxtail millet and rice [47], we used the rice genome sequence as a reference when selecting the loci so as to have wide coverage of the genome. All EST sequences of *Setaria italica *were downloaded from the NCBI ftp site http://ftp.ncbi.nlm.nih.gov. The EST set was used to search rice gene models and only those with a single hit were retained for further studies http://rice.plantbiology.msu.edu/. We checked if the homologous region covers an intron in the rice genome and designed primers to amplify the intron.

The nine loci were amplified in the two species using a modified PCR reaction system: 50 ng genomic DNA, 0.2 μmol/l of each primer, 0.2 mM dNTP, 1 U ExTaq DNA polymerase (TaKaRa), 2 μl PCR mix buffer and dH_2_O to a final volume of 20 μl. After amplification, the products were separated by electrophoresis on a 2% agarose gel. DNA bands were excised, purified, and directly sequenced on an Applied Biosystems 3730 DNA Sequencer. For individual with heterozygous alleles, It is impossible to choose a true allele if it contains more than one heterozygous polymorphism. By directly sequencing the PCR product, the haplotype that was chosen randomly may not be a true haplotype. However both foxtail millet and green foxtail have a low outcross rate, we expected that the influence of heterozygous polymorphism on haplotype inference is very limit. Single base pair changes were further confirmed by PCR and DNA sequencing.

### Sequence analysis

The raw sequence trace files were collected and assembled by Phred/Phrap [48,49]. Alleles of each locus were aligned by ClustalW 1.81 with further manual check [50]. All the alleles containing singletons were subject to a check process, in which we amplified and sequenced the product again to confirm the sequence quality and update the alignment. The alignment files were imported to DnaSP 4.5 with coding regions assigned according to the rice gene annotation [51]. For each locus and species, we calculated the number of segregating sites (S), the population recombination rate (ρ), minimum number of recombination events (Rm), number of haplotypes (Hap), haplotype diversity (Hdiv), average number of nucleotide difference per site between two sequences (π), and the watterson estimator of population mutation rate (θ). To test for neutrality, we calculated Tajima's D [52], and D* and F* of Fu and Li [53] test without outgroup. To access the level of species divergence, we calculated shared, fixed, species-specific S and Fst for the two species. Multilocus HKA test was done by HKA http://lifesci.rutgers.edu/~heylab/ for the nine loci and the results was parsed to R for further analysis [54].

The decay of linkage disequilibrium (LD) with physical distance was described using a nonlinear regression analysis. The expected value of squared allele-frequency correlations (*r*^2^) at drift-recombination equilibrium is, ^*E*(*r*^2^) = 1/(1 + *ρ*) ^where ^ρ ^is 4*Nc *and *N *is the effective population size, *c *is the recombination rate in Morgans between the 2 markers. Under the assumption of a low mutation rate and finite sample size, the expectation becomes

$$E\left(r^{2}\right)=\left[\frac{10+\rho }{\left(2+\rho \right)\left(11+\rho \right)}\right]\left[1+\frac{\left(3+\rho \right)\left(12+12\rho +\rho^{2}\right)}{n\left(2+\rho \right)\left(11+\rho \right)}\right]$$

where n is the sample size of sequences [55]. To introduce the distance between pairs of SNP sites (d) into the formula, we use rho*d to replace ρ, in which rho is the recombination rate per basepair. *r*^2 ^between pairs of polymorphism was estimated using DnaSP 4.5 for each locus and species. *r*^2 ^and d were pooled across the loci for each species. The nonlinear regression analysis was performed with the NLS function in the R statistical package http://www.r-project.org.

### Coalescent simulation

Coalescent simulation was used to model the process of domestication, as well as to estimate the population mutation rate θ and population recombination rate ρ for each species. The simulations were done using Hudson's *ms *[56]. In each simulation, we used an rejection-based approximate Bayesian computation approach to obtain a posterior distribution for parameters of interest [57,58]. Briefly, the initial values of parameters were drawn from a user-specified prior distribution, and, starting with these parameters, the simulation was run under a defined model; For each simulated datasets, several summary statistics were calculated and compared with the observed values. The data was accepted if it was within a defined interval of observed data; and then the parameter set that generated acceptable data were used to obtain a posterior distribution for each parameter. In this study, we used a multilocus approach to assess the acceptability of the data. Summary statistics were calculated for each locus and summarized by mean and/or variance across the loci. The values of mean and/or variance of each summary statistics were compared between simulated and observed data using. The accepted datasets were used for Baysian inference of selceted parameters. Doing this incorporates the variation among the loci into the simulations.

### Approximate Bayesian estimate of θ and ρ/θ

To estimate θ and ρ, the simulations were run under the standard neutral model for the wild and domesticated species separately. The means for each four summary statistics (S, π, Hap, Hdiv) were used to access the acceptable of the simulated data. The data was accepted if three of them were within 20% of the observed data [59].

### Approximate Bayesian inference of bottleneck parameters

The model used to investigate the bottleneck process was similar as described for maize and rice [3,6,45]: *N_a _*is the effective population size of the ancestor of the two species.μis the mutation rate for the ancestor. The values of μ were based on the synonymous substitution rate or calculated by θ = 4*N*μ. The recombination rate ρ together with *N_a _*and μ defined the ancestor population. At time t2 generations ago, a new population was derived from the ancestor with a population size of *N_b _*and expanded to a population with a size of *N_p _*at t1 generations ago. Then, the ancestor population and the newly derived population evolved with constant population size until present. To incorporate gene flow in this model, we defined M12 to be migration rate from wild to cultivar and M21 for the rate for the reverse rate. In this model, the parameters for the wild and domesticated species were calculated by the sequence or estimated using an approximate Bayesian estimate. We defined k to be the ratio of *N_b _*and d, d being the duration of the bottleneck in generations (d = t2-t1). Previous studies suggested that *N_b _*is positively correlated with d; k is a good indicator for the stringency of the bottleneck [3,6].

Under this model, we used the mean and variance for each four summary statistics (S, π, Hap, Hdiv) to assess the acceptability of simulated wild species data. The simulation was accepted if both the mean and variance fell within 30% of the observed data. To fit the cultivated data, we use the mean of S, ρ, Hap, and Hdiv to assess the fitness of the simulated data, but we accepted if three of the four fell within 30% of the observed data.

## Authors' contributions

XD and MC designed the study, supervised the experiment. HZ and WL collected and planted the samples used and made help in laboratory work. CW, JC, LY, HL and YW designed primers and carried out the laboratory work. JC, BZ, CW and XD performed the statistical analysis. JC prepared the first draft of the manuscript and XD made optimization of the manuscript. All authors discussed the result and conclusion and read and approved the final manuscript.

## Supplementary Material

Additional file 1 **Schematic diagrams of nine loci and sequenced regions in this study**. Exons, introns and UTRs are indicated by blue boxes, lines and open boxes. The primers that were used to PCR and sequencing are marked with black arrowhead, where F and R stand for forward primer and reverse primer respectively. Click here for file
