# Supplementary figures and images for: Population genetics of foxtail millet and its wild ancestor
Source: BMC Genet. 2010 Oct 11;11:90. doi: 10.1186/1471-2156-11-90 (PMC2964552; doi:10.1186/1471-2156-11-90)

Figure S1

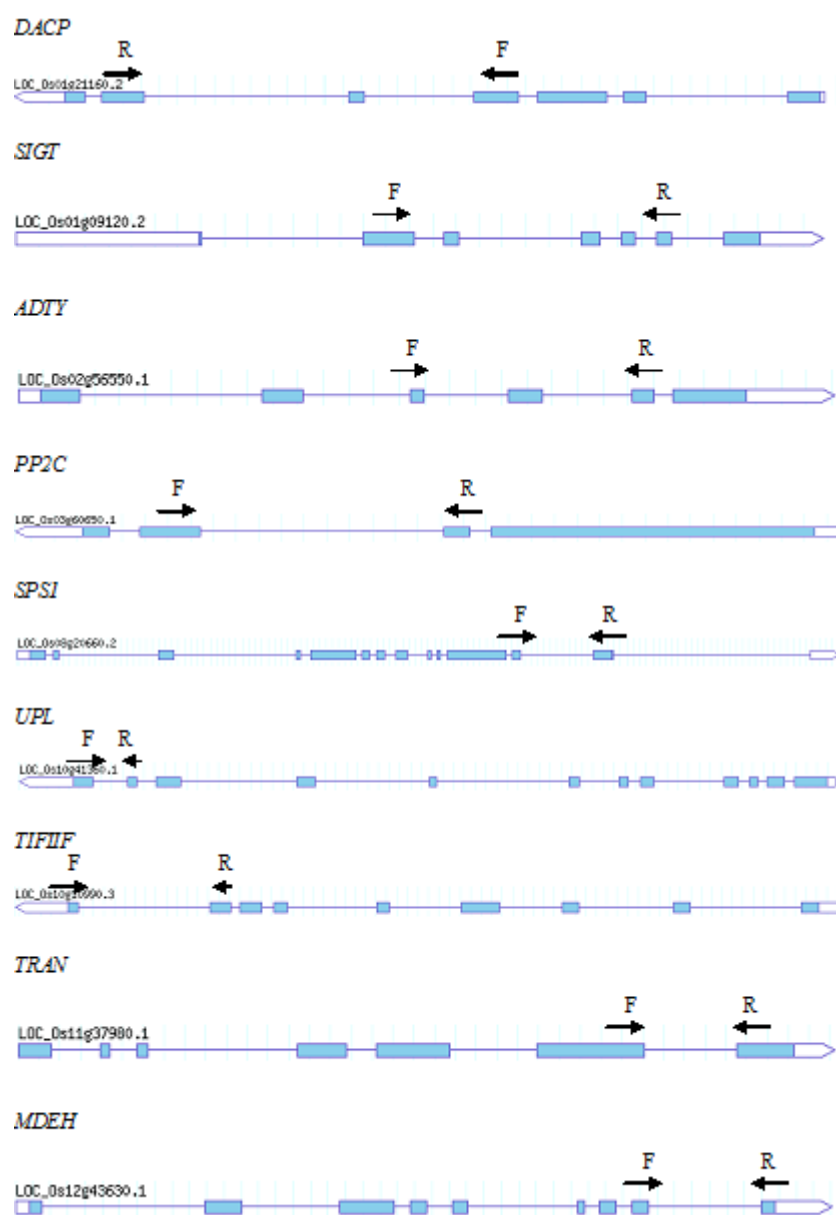

Supplement: Additional file 1 — Schematic diagrams of nine loci and sequenced regions in this study. Exons, introns and UTRs are indicated by blue boxes, lines and open boxes. The primers that were used to PCR and sequencing are marked with black arrowhead, where F and R stand for forward primer and reverse primer respectively. [file 1471-2156-11-90-S1.PDF]
